# Immunogenicity and Protective Efficacy of Psoralen-Inactivated SARS-CoV-2 Vaccine in Nonhuman Primates

**DOI:** 10.3390/vaccines12050451

**Published:** 2024-04-24

**Authors:** John W. Sanders, Daniel Ewing, Appavu K. Sundaram, Christopher Scott Gamble, Maria Blevins, Zhaodong Liang, Leigh Ann Sanders, David A. Ornelles, Peifang Sun, Klara Lenart, Hendrik Feuerstein, Karin Loré, Nikolai Petrovsky, Maya Williams, Kevin R. Porter

**Affiliations:** 1 Section on Infectious Diseases, Wake Forest University School of Medicine, Winston-Salem, NC 27157, USA; jwsander@wakehealth.edu (J.W.S.); csgamble@wakehealth.edu (C.S.G.); ornelles@wakehealth.edu (D.A.O.); 2 Agile Vaccines and Therapeutics Department, Naval Medical Research Command, Silver Spring, MD 20910, USA; 3 Henry M. Jackson Foundation, Rockledge Drive, Bethesda, MD 20817, USA; 4 Department of Medicine Solna, Division of Immunology and Allergy, Karolinska Institutet and Karolinska University Hospital, 17177 Stockholm, Sweden; 5 Center for Molecular Medicine, Karolinska Institutet, 17177 Stockholm, Sweden; 6 Vaxine Pty Ltd., Warradale, SA 5046, Australia; 7 United States Navy Bureau of Medicine and Surgery, Frederick, MD 21702, USA; 8 Directorate for Defense Infectious Diseases Research, Naval Medical Research Command, Silver Spring, MD 20910, USA; kevin.r.porter3.civ@health.mil

**Keywords:** SARS-CoV-2, COVID-19, SARS-CoV-2 PsIV, SARS-CoV-2 neutralizing antibodies, conformational epitopes, Advax-CpG, DNA vaccine

## Abstract

COVID-19 caused by severe acute respiratory syndrome coronavirus 2 (SARS-CoV-2) has significantly impacted public health and the economy worldwide. Most of the currently licensed COVID-19 vaccines act by inhibiting the receptor-binding function of the SARS-CoV-2 spike protein. The constant emergence of SARS-CoV-2 variants resulting from mutations in the receptor-binding domain (RBD) leads to vaccine immune evasion and underscores the importance of broadly acting COVID-19 vaccines. Inactivated whole virus vaccines can elicit broader immune responses to multiple epitopes of several antigens and help overcome such immune evasions. We prepared a psoralen-inactivated SARS-CoV-2 vaccine (SARS-CoV-2 PsIV) and evaluated its immunogenicity and efficacy in nonhuman primates (NHPs) when administered with the Advax-CpG adjuvant. We also evaluated the SARS-CoV-2 PsIV as a booster shot in animals vaccinated with a DNA vaccine that can express the full-length spike protein. The Advax-CpG-adjuvanted SARS-CoV-2 PsIV elicited a dose-dependent neutralizing antibody response in the NHPs, as measured using a serum microneutralization assay against the SARS-CoV-2 Washington strain and the Delta variant. The animals vaccinated with the DNA vaccine followed by a boosting dose of the SARS-CoV-2 PsIV exhibited the highest neutralizing antibody responses and were able to quickly clear infection after an intranasal challenge with the SARS-CoV-2 Delta variant. Overall, the data show that the Advax-CpG-adjuvanted SARS-CoV-2 PsIV, either by itself or as a booster shot following nucleic acid (NA) vaccines, has the potential to protect against emerging variants.

## 1. Introduction

Within the last two decades, three different coronaviruses with pandemic potential have crossed species barriers and infected humans, resulting in serious respiratory illness and death [1,2,3]. COVID-19 emerged in 2019 and has since spread rapidly throughout the world, resulting in global economic hardships and strain on healthcare systems worldwide. Although most SARS-CoV-2 infections result in a mild form of COVID-19, with patients recovering within 3 weeks, a sizable number of patients proceed to severe COVID-19 disease, with increased fatality. Globally, as of 30 November 2023, more than 772 million confirmed COVID-19 cases and over 6.9 million COVID-19-related deaths have been reported [4]. Indeed, with SARS-CoV-2 nucleocapsid protein seroprevalence in many countries in the range of 80–90%, it is likely that the vast majority of the global population has by now been infected by SARS-CoV-2 [5].

As a result of the global priority to protect against COVID-19, more than 10 different COVID-19 vaccines developed using various platforms are now available for human use [6]. The majority of the COVID-19 vaccines currently in use or in various stages of development are designed to elicit immune responses by targeting the spike protein receptor-binding domain (RBD) and include viral vector-based vaccines, recombinant protein vaccines and the recently developed messenger RNA vaccines [3,7]. Although these vaccines targeting the spike protein alone show good short-term protective efficacy against COVID-19, their long-term protective efficacy is still not known, especially due to the emergence of SARS-CoV-2 variants with mutations in the RBD [8]. In addition, the effectiveness of the current vaccines against newly emerging variants is also a concern, as the Omicron variant has shown a higher rate of infectivity than wild-type SARS-CoV-2 or the Delta variant, albeit causing less severe disease [8]. Additional mutations could lead to the emergence of variants with higher transmissibility and the ability to cause severe disease. Therefore, the development of a whole-virus inactivated vaccine that mimics natural infection in terms of presenting several viral antigens to the host immune system to elicit a broader immune response against variants of concern is warranted [3,9,10,11,12,13]. Although β-propiolactone-inactivated SARS-CoV-2 vaccines have been prepared and are in use currently, the efficacy of these vaccines are less than optimal since β-propiolactone can alter the antigenic conformations via protein degradation during the inactivation process [14,15]. β-propiolactone and formalin inactivate viruses by acting on both proteins and nucleic acids. They are also hazardous chemicals that should be handles with extreme caution, as they are considered carcinogenic. Psoralen compounds on the other hand intercalate with nucleic acids and, upon irradiation with long-wavelength ultraviolet (UV) light, form a covalent bond with pyrimidine bases on adjacent strands, resulting in inter-strand cross-links that prevent replication [3,16,17,18]. Therefore, psoralen compounds inactivate viruses only at the nucleic acid level and presumably preserve the antigenic conformational epitopes on the virus surface. We have previously performed psoralen-inactivation of all four serotypes of dengue virus and prepared psoralen-inactivated dengue virus vaccines. Monovalent and tetravalent psoralen-inactivated dengue vaccines formulated with an adjuvant such as Advax-CpG (VO_0005324 (https://vac.niaid.nih.gov/view?id=38 (accessed on 1 April 2024)) were shown to be immunogenic in mice and nonhuman primates [19]. Recently, we prepared psoralen-inactivated SARS-CoV-2 (SARS-CoV-2 PsIV) and evaluated its immunogenicity in mice [3].

Here, we describe the chromatographic purification of the SARS-CoV-2 PsIV and the immunogenicity and protective efficacy of the purified SARS-CoV-2 PsIV in nonhuman primates (NHPs) using 10^8^–5 × 10^10^ particles/dose combined with the Advax-CpG adjuvant. We also describe the results of SARS-CoV-2 PsIV’s evaluation as a booster shot in animals vaccinated with a DNA vaccine expressing the SARS-CoV-2 spike glycoprotein.

## 2. Materials and Methods

### 2.1. Chemicals and Reagents

The 4′-aminomethyl-4,5′,8-trimethylpsoralen (AMT) was purchased from Cayman Chemical (Ann Arbor, MI, USA). Recombinant human serum albumin was purchased from eENZYME, LLC (Gaithersburg, MD, USA). The Pluronic F-127 co-polymer was purchased from Sigma-Aldrich (St. Louis, MO, USA). The Advax-CpG55.2 adjuvant was provided by Vaxine Pty, Ltd., Warradale, Australia. A rabbit antibody against the SARS-CoV-2 spike RBD (Catalog# 40592-T62, Sino Biological, (Wayne, PA, USA)) was used as the primary antibody for the ELISA. A goat anti-rabbit IgG–horseradish peroxidase (HRP) conjugate (catalog#31460, Thermo Fisher Scientific (Waltham, MA, USA)) was used as the secondary antibody for the ELISA. ABTS One Component (catalog# 5120-0043, SeraCare Inc. (Gaithersburg, MD, USA)) was used as the substrate. The SARS-CoV-2 spike protein quantitative ELISA kit (cat# KIT40591) was purchased from Sino Biological US Inc (Wayne, PA, USA). Western blot analysis of SARS-CoV-2 was performed using an anti-nucleocapsid protein Abs (catalog# 9103, ProSci, Inc. (Fort Collins, CO, USA)), an anti-spike Abs (catalog# 3525, ProSci, Inc. (Fort Collins, CO, USA)) and an anti-membrane protein Abs (catalog# 10-516, ProSci, Inc. (Fort Collins, CO, USA)) as the primary antibodies and a goat anti-rabbit IgG–HRP conjugate (catalog#31460, Thermo Fisher Scientific (Waltham, MA, USA)) as the secondary antibody. A rabbit anti-spike RBD Ab (catalog# 40592-T62, Sino Biological (Wayne, PA, USA)) and a goat anti-rabbit IgG fluorescein-conjugated Ab (catalog# 611-102-122, Rockland Immunochemicals Inc. (Pottstown, PA, USA)) were used as the primary and secondary antibodies, respectively, for the immunofluorescence assays (IFAs). All the other chemicals and reagents were purchased from Thermo Fisher Scientific (Waltham, MA, USA).

### 2.2. Preparation of SARS-CoV-2 PsIV

SARS-CoV-2 was propagated, harvested and inactivated as described previously [3]. The SARS-CoV-2 strain nCoV/USA-WA1/2020 was propagated in Vero E6 cell cultures and harvested through centrifugation at 3000× *g* for 15 min. Two liters of culture supernatant containing the SARS-CoV-2 particles was then treated with benzonase to remove the host cell DNA, and then the volume was reduced to 100 mL (concentrating) and the buffer exchanged with 10 mM tris buffer containing 150 mM sodium chloride using 100 K molecular weight cut-off (MWCO) membrane filter cassettes (catalog# VF20P4, Sartorius (Bohemia, NY, USA)). The concentrated SARS-CoV-2 virus preparation was mixed with AMT (30 µg of AMT per 1 mL of virus), and the resulting virus/AMT mixture was UV-irradiated (365 nm) for 5 min (the total energy applied to the SARS-CoV-2/AMT mixture was 1,445,400 µjoules). The absence of a viable virus in the inactivated virus suspension was confirmed through its inability to grow in permissive cells (Vero E6 cells) using a virus amplification test, as reported previously [3]. Briefly, 50 µL aliquots of the inactivated virus was used to infect cultured cells in duplicate, and they were incubated at 37 °C for 5–8 days. After the incubation, the cells and culture supernatants were examined for the presence of SARS-CoV-2 antigens using indirect immunofluorescence assay and Western blot analysis, respectively. The supernatant from this first culture (50 µL aliquot) was then used to infect fresh Vero E6 cells for a second round of amplification and testing. Negative results (indicating the absence of SARS-CoV-2 antigens) in both the passages confirmed the complete inactivation of SARS-CoV-2.

### 2.3. Purification and Characterization of the SARS-CoV-2 PsIV

The SARS-CoV-2 PsIV was purified using chromatographic methods using Cellufine MAX DexS-VirS resin (catalog# 21 701 JNC America, Inc. (Burlingame, CA, USA)). Briefly, the SARS-CoV-2 PsIV in 10 mM tris buffer containing 150 mM sodium chloride was passed through a 25 mL Max DexS-VirS pack in a XK 16/40 column (catalog# 2898838 Cytiva (Marlborough, MA, USA)) at 0.5 mL per minute, followed by washing with 50 mL of 10 mM tris buffer containing 150 mM sodium chloride. The SARS-CoV-2 PsIV bound to the column resin was then eluted using 500 mM sodium chloride in 10 mM tris buffer. The fractions containing the SARS-CoV-2 PsIV, eluted using 500 mM sodium chloride and identified using Western blot analysis with a SARS-CoV-2-specific anti-spike protein antibody, were then pooled together as the purified SARS-CoV-2 PsIV. This material was further purified, and the buffer was exchanged with PBS using 25 mL of a dual-mode Capto Core 700 resin (catalog# 17548104 Cytiva (Marlborough, MA, USA)) packed in a XK 16/40 column (catalog# 2898838 Cytiva (Marlborough, MA, USA)).

The pure SARS-CoV-2 PsIV was then mixed with stabilizer solution containing Pluronic F-127 (2%), trehalose (15%) and recombinant human serum albumin (0.5), sterile-filtered using a 0.22 micron filter and stored at −80 °C until further use [3]. The purified inactivated virus preparation was tested using Western blot assays to confirm the presence of SARS-CoV-2-specific spike, nucleoprotein and envelope proteins. The purity of the SARS-CoV-2 PsIV was confirmed using gel electrophoresis, followed by silver staining to detect any contaminating proteins. The SARS-CoV-2 PsIV titer was determined using a quantitative ELISA for the spike protein using the SARS-CoV-2 (2019-nCoV) Spike ELISA Kit (cat# KIT40591, Sino Biological US Inc. (Wayne, PA, USA)). The vaccine doses for the NHP study were prepared based on the spike protein concentration and particle numbers, which we calculated based on the molecular weight of the spike protein (approximately 150 kDa) and the estimated average of 300 copies of spike protein per virus particle.

### 2.4. SARS-CoV-2 DNA Vaccine

The DNA sequences encoding the spike protein of SARS-CoV-2 strain nCoV/USA-WA1/2020 were synthesized and cloned into the plasmid vector VR1012 (Vical, Inc. (San Diego, CA, USA)), as reported previously [3]. Purified endotoxin-free recombinant DNA constructs were prepared and used as the DNA vaccine candidate.

### 2.5. Evaluation of Immunogenicity in Nonhuman Primates

**Figure 1:**
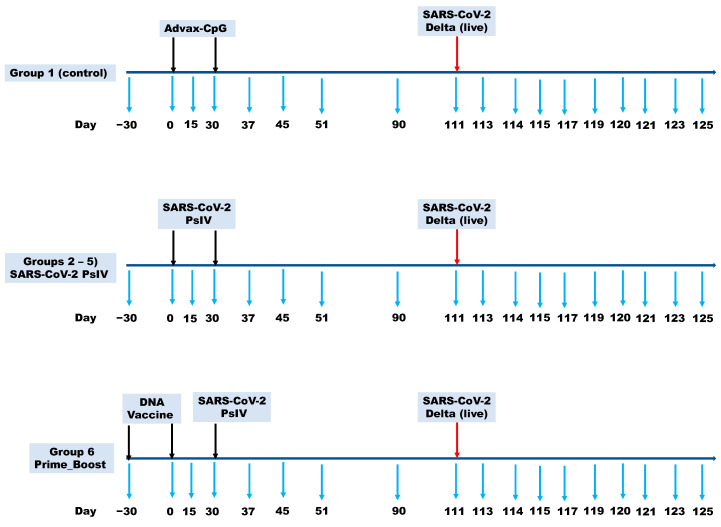
NHP vaccination and sample collection (blood draws for sera and PBMC preparation, BAL samples and nose and throat swabs) schedule. Black arrows indicate the vaccination schedule. Red arrow indicates challenging the animals with live virus. Blue arrows indicate the sample collection schedule.

The experiments reported herein were conducted in compliance with the Animal Welfare Act and in accordance with the principles set forth in the Guide for the Care and Use of Laboratory Animals, National Research Council, National Academy Press, 1996. The study protocol (#A20-141) was reviewed and approved by the Wake Forest University Institutional Animal Care and Use Committee (IACUC) and the U.S. Navy Bureau of Medicine and Surgery (BUMED) in compliance with all the applicable federal regulations governing the protection of animals and research. The SARS-CoV-2 PsIV alone and in a prime-boost regimen, using two doses of the DNA vaccine followed by boosting with the SARS-CoV-2 PsIV, was evaluated for its immunogenicity in NHPs (Cynomolgus macaques), as shown in Table 1. Five groups of 8 animals each were immunized with two doses of different amounts of the SARS-CoV-2 PsIV with 10 mg of the Advax-CpG adjuvant on days 0 and 30. The animals in group 1 (control group) received the Advax-CpG adjuvant in PBS on days 0 and 30. The animals in groups 2–5 received different amounts of the SARS-CoV-2 PsIV (0.0075–3.75 µg of spike protein equivalent per dose) with the Advax-CpG adjuvant on days 0 and 30. The animals in group 6 (prime-boost group) received the DNA vaccine on days −30 and 0, followed by a boost with the SARS-CoV-2 PsIV (3.75 µg spike protein equivalent) on day 30. The SARS-CoV-2 PsIVs were administered via intramuscular injection (IM) using a PharmaJet Stratis needle-less injector. The DNA vaccine (encoding the SARS-CoV-2 spike protein) was administered via the IM route using the Ichor electroporation device. Blood was collected from all the animals on days −30, 0, 30, 51, 90 and 120, and the serum preparations were tested for the presence of neutralizing antibodies against SARS-CoV-2 using the microneutralization assays. Blood was also drawn from all the animals and processed to obtain their PBMCs on day −30 (baseline PBMCs) and on day 45 (15 days after the last dose of the vaccine) (Figure 1).

On day 95, four animals from each of groups 3–6 and four animals from the control group (total of 20 animals) were moved to the ABSL-3 facility to acclimate in preparation for challenge with the live SARS-CoV-2 virus. On day 111, these twenty animals were challenged with 2 × 10^5^ PFU of the SARS-CoV-2 Delta strain at a dose of 0.5 mL via intranasal instillation (1 × 10^5^ PFU per nostril). Blood was collected from all the challenged animals on days 111 and 126. Nasal swabs and throat swabs were also collected from the challenged animals on alternate days, from day 111 to day 125. Bronchoalveolar lavage (BAL) samples were collected from the challenged animals on days 111, 114, 117 and 120.

### 2.6. SARS-CoV-2 Microneutralization Assay

The serum neutralizing antibody levels against SARS-CoV-2 were determined using a microneutralization test against the Washington strain and the Delta variant, as published previously [3]. Briefly, 200 TCID_50_ of the SARS-CoV-2 test strain was incubated with two-fold dilutions of the serum samples in 96-well plates for 1 h at 37 °C. Vero 81 cells (2 × 10^4^) were then added to each well and incubated at 37 °C for 84 h. After 84 h, the cells were fixed by adding 200 µL of cold fixation solution (1:1 mixture of ethanol and methanol) to each well and incubating the plates at −20 °C for 30 min. Once the cells were fixed, the SARS-CoV-2 titer in each well was measured by quantitating the spike protein using a SARS-CoV-2-specific anti-S antibody in a standard ELISA format, and the absorbance at 405 nm was measured using a BioTek Epoch plate reader (Agilent Technologies Inc. (Wood Dale, IL, USA)). The highest serum dilution that resulted in a ≥80% reduction in absorbance when compared to the control samples (without neutralizing antibodies) was determined as the 80% microneutralization titer (MN_80_).

### 2.7. ELISA

The binding antibody titers using ELISA were assessed according to methods previously published [20]. Briefly, 96-well half-area ELISA plates (Corning (Tewksbury, MA, USA)) were coated with the recombinant Washington strain (WA-1) spike protein (expressed as a prefusion-stabilized S-2P construct) [21] at 2 μg/mL in PBS, followed by overnight incubation at 4 °C. The next day, the plates were washed three times with PBS-T (PBS containing 0.05% Tween 20) and blocked with 5% milk (Oxoid skim milk powder, Thermo Scientific, Waltham, MA, USA) in PBS (*w*/*v*) for 1 h at room temperature. Serially diluted plasma samples in duplicate were added to the plates and incubated for 2 h at room temperature. The plates were washed three times, and a goat anti-monkey IgG–horseradish peroxidase secondary antibody (Nordic-MUBio (Susteren, the Netherlands)) was added for 1 h at room temperature. The plates were washed three times again, and 1-Step Ultra TMB-ELISA substrate (Thermo Fisher Scientific, Waltham, MA, USA) was added for 5 min. The reaction was stopped with 1 M H_2_SO_4_, and the absorbance was measured at 450 nm with background correction at 570 nm. The data were analyzed using Prism v9.4.1 using a 4-parameter logistic curve fit.

### 2.8. Memory B Cell Assay

To generate the protein probes, the recombinant WA-1 S-2P and RBD proteins were biotinylated using the EZ-Link Micro Sulfo-NHS-LC Biotinylation Kit (Thermo Fisher Scientific, Waltham, MA, USA) according to the manufacturer’s instructions [20]. Streptavidin-conjugated fluorophores (SA-PE, SA-APC or SA-BV421) and biotinylated proteins were coupled at a 4:1 molar ratio. The cryopreserved PBMCs were thawed and stained with 100 ng of the fluorescent protein probes for 20 min at 4 °C, followed by staining with 7-aminoactinomycin D (7-AAD, Thermo Fisher, Waltham, MA, USA) and a panel of antibodies, IgM PerCP-Cy5.5 (G20-127, BD, Franklin Lakes, NJ, USA), IgD FITC (polyclonal, Southern Biotech, Birmingham, AL, USA), CD3 BV510 (SP34-2, BD), CD14 BV510 (M5E2, BioLegend, San Diego, CA, USA), CD16 BV510 (3G8, BD), CD20 BV605 (2H7, BioLegend, San Diego, CA, USA), HLA-DR BV650 (L243, BioLegend, San Diego, CA, USA) and IgG BV786 (G18-145, BD, Franklin Lakes, NJ, USA), for another 20 min at 4 °C. After staining, the cells were washed with FACS buffer (PBS supplemented with 2% heat-inactivated fetal calf serum) and fixed with 1% formaldehyde solution. The samples were acquired using a BD LSRFortessa cell analyzer (BD, Franklin Lakes, NJ, USA). The data were analyzed using FlowJo software v.10.7.1 (FlowJo, Ashland, OR, USA).

### 2.9. T Cell Recall Assay

Spike-specific memory T cells in the blood were evaluated using a recall assay using 2 μg/mL of PepMix SARS-CoV-2 spike overlapping peptides in DMSO (15 mers with 11-amino-acid overlap, JPT Peptide Technologies), with an equimolar amount of DMSO as the negative control or Staphylococcal enterotoxin B as the positive control, as described previously [20]. The cells were cultured overnight at 37 °C and 5% CO_2_ in the presence of 10 μg/mL Brefeldin A (Thermo Fisher, Waltham, MA, USA). The next day, the cell cultures were washed with PBS and stained with LIVE/DEAD blue dye (Thermo Fisher, Waltham, MA, USA) and the following panel of antibodies: CD103 FITC (2G5, Beckman Coulter (Brea, CA, USA)), CD4 PE-Cy5.5 (S3.5, Invitrogen (Carlsbad, CA, USA)), CCR7 BV421 (G043H7, BioLegend (San Diego, CA, USA)), CD45RA BV650 (5H9, BD, Franklin Lakes, NJ, USA) and CD8a BV711 (RPA-T8, BioLegend, (San Diego, CA, USA), Following permeabilization with a Cytoperm/Cytofix fixation kit (BD, Franklin Lakes, NJ, USA), the cells were stained for the following intracellular proteins: CD69 ECD, TP.1.55.3( Beckman Coulter, Sykeville, MD, USA), IL-13 PE (JES10-5A2, BD, Franklin Lakes, NJ, USA), CD3 APC-Cy7 (SP34-2, BD, Franklin Lakes, NJ, USA), IFNγ AF700 (B27, BioLegend, (San Diego, CA, USA), IL-21 AF647 (3A3-N2.1, BD, Franklin Lakes, NJ, USA), IL-2 BV605 (MQ1-17H12, BD, Franklin Lakes, NJ, USA) and IL-17A BV785 (BL168, BioLegend, (San Diego, CA, USA). The samples were acquired using the BD LSRFortessa cell analyzer (BD, Franklin Lakes, NJ, USA, and the data were analyzed using FlowJo software v.10.7.1 (FlowJo (Ashland, OR, USA).

### 2.10. Quantitative ELISA for Domain-Specific IgG

For the ELISA coating antigens, the RBD was kindly provided by Dr. Le Jiang. All the other antigens were commercially obtained from ACROBiosystems (Newark, DE, USA): RBD Delta (SPD-C52Hf), RBD Omicron (SPD-C522e), NTD Ancestral (S1D-C52H6), NTD Delta (S1D-C52Hf), NTD Omicron BA.1 (SPD-C522d), S1 Ancestral (S1N-C52H3), S1 delta (S1N-C52Ha) and S1 Omicron BA.1 (S1N-C52Ha). Phosphate-buffered saline (PBS) was used as the coating buffer; PBS with 0.1% Tween 20 (PBST) was used as the wash buffer; five percent Difco skim milk in PBST was used as the blocking buffer and sample dilution buffer; Immulon 4 HBX 96-well, flat-bottom microplates (Thermo; Cat: 3855 (Waltham, MA, USA)) were used as the assay plates. Prior to the immunoassay, the serum samples were heat-inactivated at 56 °C for 1 h.

For the IgG standard curves, the microplates were coated with 1 µg/mL (0.1 µg/well) of goat anti-human IgG (Southern Biotech (Birmingham, AL, USA)) in coating buffer at 4 °C overnight. After washing them with PBST, the plates were blocked with blocking buffer at 37 °C for 1 h. Purified IgG (Athens Research, Catalog# 16-16-090707 (Athens, GA, USA)) was 2-fold serially diluted with the sample dilution buffer and used as the standard (ranging from 0.4 ng/mL to 800 ng/mL). The plates were then incubated at 37 °C for 1 h. After washing them with PBST, the plates were incubated with 1:1000-diluted goat anti-human IgG (H+L)–HRP (KPL, Cat:474-1006 (Gaithersburg, MD, USA)) at 37 °C for 1 h. HRP was detected using the TMB Microwell Peroxidase substrate, and the reactions were stopped by adding 1N sulfuric acid (H_2_SO_4_) solution. The plates were then read at OD 450 nm using a plate reader (PerkinElmer EnSpire (Waltham, MA, USA)) within 30 min of stopping the reaction.

For the SARS-CoV-2-specific IgG ELISA, the microplates were coated with 1 µg/mL (0.1 µg/well) of recombinant proteins in coating buffer at 4 °C overnight. After washing them with PBST, the plates were blocked with blocking buffer at 37 °C for 1 h. The serum samples were diluted either 1:100 or 1:1000 in the sample dilution buffer and incubated on plates at 37 °C for 1 h. The same ELISA settings with the coating buffer were used to detect and subtract the background signal for each sample. SARS-CoV-2-specific IgG detection and development were performed as described above. The IgG concentrations (ng/mL) of each sample were calculated according to their sigmoidal standard curves using GraphPad Prism 9.0.2 (Boston, MA, USA).

Convalescent sera obtained from Biodefense and Emerging Infections (BEI) Resources (Manassas, VA, USA) were used as the reference sera. Deidentified human sera obtained from a previous study, Survey of Immune Response to Coronavirus Disease 2019 Infections (SIM-COVID), where the samples were from subjects vaccinated with 2 doses of the Moderna RNA vaccine and the samples were collected within 1 month from vaccination (referred to as SIM/Moderna sera in this article), were used as the positive control samples.

### 2.11. Digital Droplet RT-PCR

The absolute copy number of SARS-CoV-2 RNA in the NHP samples was measured using digital droplet reverse transcriptase–polymerase chain reaction (RT-ddPCR) [22,23]. The RNA from the NHP samples was extracted using the Direct-zol-96 kit (catalog#R2054, Zymo Research, inc. (Irvine, CA, USA)). A total of 100 µL of the NHP samples was mixed with 300 µL of the TRIzol reagent from the kit, and the RNA from the TRIzol-treated samples was extracted using a KingFisher Flex automated magnetic particle processor (Thermo Fisher Scientific (Waltham, MA, USA)). SARS-CoV-2 RNA quantitation in the extracted nucleic acid samples was performed using the One-Step RT-ddPCR Advanced Kit for Probes (Catalog# 1864022, Bio-Rad (Des Plaines, IL, USA)) and SARS-CoV-2-nucleocapsid-specific N1 primers and probes. The primer sequences targeted the nucleocapsid genes of SARS-CoV-2. The forward and reverse primer sequences were 5′-GACCCCAAAATCAGCGAAAT-3′ and 5′-TCTGGTTACTGCCAGTTGAATCTG-3′, respectively. The probe sequence used was 5′-ACCCCGCAT-/ZEN/-TACGTTTGGTGGACC-3′-3IABkFQ. Briefly, RT-ddPCR droplets were made by mixing 20 µL of the RT-ddPCR reaction mixture with 70 µL of automated droplet generation oil for probes (catalog# 1864110, Bio-Rad (Des Plaines, IL, USA)) using an automated Droplet Generator (Bio-Rad (Des Plaines, IL, USA)). After the completion of the thermocycling, the fluorescence intensity in each droplet was read using a QX200 Droplet Reader (Bio-Rad (Des Plaines, IL, USA)), and the data were analyzed using QuantaSoft software v1.4 (Bio-Rad (Des Plaines, IL, USA). The ddRT-PCR results are expressed as the total number of RNA copies.

### 2.12. Data Analysis

The data analysis was performed using GraphPad Prism 9.0.2 or 9.4.1. Student’s *t*-tests were used to compare the microneutralization data between groups. Statistical significance is indicated as * *p* < 0.05, ** *p* < 0.01, *** *p* < 0.001 and **** *p* < 0.0001. Statistical comparison of the IgG antibody values, memory T cell values and memory B cell values between groups was performed using the Kruskal–Wallis test for each timepoint. *p*-values were adjusted for multiple testing using Dunn’s multiple comparison test.

## 3. Results

### 3.1. Preparation and Purification of the SARS-CoV-2 PsIV

**Figure 2:**
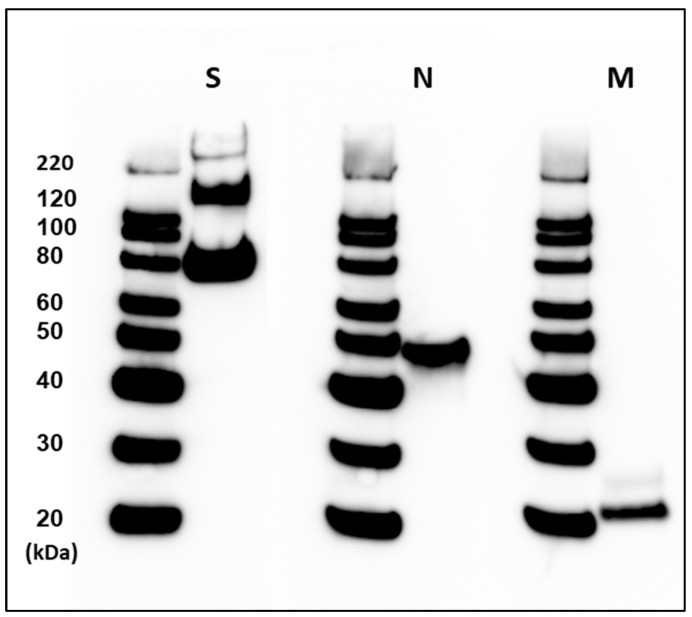
Western blot analysis of SARS-CoV-2 PsIV after two-step chromatographic purification using Cellufine MAX-DexS-VirS column and a Capto Core 700 column. Presence of SARS-CoV-2 spike glycoprotein (S), nucleocapsid protein (N) and membrane protein (M) was confirmed by binding to the respective antibodies. Intensities of S, N and M bands relative to the 80 KDa molecular weight marker intensity are provided in the Supplementary Materials.

We obtained a highly purified SARS-CoV-2 PsIV at a concentration of 7.5 µg of spike protein per mL (equivalent to 10^11^ particles of SARS-CoV-2/mL) after a two-step chromatographic purification method using a MAX DexS-VirS column and a Capto Core 700 column. Western blot analysis of the purified SARS-CoV-2 PsIV using anti-spike-, anti-nucleocapsid- and anti-envelope-protein-specific antibodies confirmed the presence of SARS-CoV-2 structural protein antigens (Figure 2). The SARS-CoV-2 PsIV was then mixed with Advax-CpG to obtain vaccines with titers ranging from 0.0075 µg to 3.75 µg of spike protein (equivalent to 10^8^–5 × 10^10^ particles of SARS-CoV-2) and 10 mg of Advax-CpG per dose in one mL.

### 3.2. Immunogenicity of the SARS-CoV-2 PsIV in Nonhuman Primates

**Figure 3:**
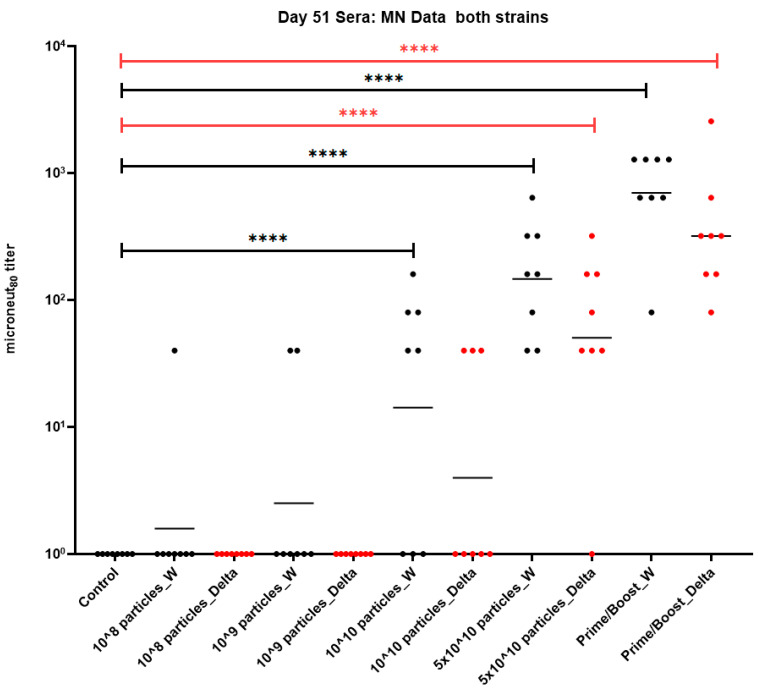
MN_80_ data (day 51 sera) from nonhuman primates vaccinated with SARS-CoV-2 PsIV. Data (average of duplicate results) for individual animals are represented by circles, and the geometric mean titer for each group is represented by a horizontal bar. Statistical significance between groups is denoted by **** *p* ≤ 0.0001.

Five groups each containing eight NHPs (Cynomolgus macaques) were immunized with PBS (control group) or 0.0075 µg–3.75 µg of spike protein per dose (equivalent to 10^8^–5 × 10^10^ particles per dose) of the SARS-CoV-2 PsIVs formulated with the Advax-CpG adjuvant on days 0 and 30, as shown in Table 1. A sixth group of eight animals (Prime-boost group) was vaccinated with two doses of the DNA vaccine encoding the SARS-CoV-2 spike protein on days −30 and 0 followed by a third dose of the SARS-CoV-2 PsIV (5 × 10^10^ particles per dose) on day 30. Their blood was collected on day −30 (only the prime-boost group) and from all animals on days 0, 30, 35 and 51. Their serum was tested for the neutralization of the SARS-CoV-2 Washington strain and the Delta variant using microneutralization assays. All the microneutralization data are reported as MN_80_ titers, reflecting an 80% reduction in the infection of the cells in vitro when compared to the level of infection in the absence of neutralizing antibodies. The MN_80_ data for the day 51 sera (three weeks after the administration of the last dose) are shown in Figure 3.

Lower doses of the SARS-CoV-2 PsIV (10^8^ and 10^9^ particles/dose) did not induce any detectable SARS-CoV-2 neutralizing antibody responses. However, higher doses of the SARS-CoV-2 PsIV (10^10^ and 5 × 10^10^ particles/dose) induced measurable neutralizing antibody levels against both the Washington and Delta strains (Figure 3). The animals vaccinated with 5 × 10^10^ particles per dose of the SARS-CoV-2 PsIV elicited a neutralizing antibody response against both the Washington and Delta strains with MN_80_ geometric mean titers (GMTs) of 147 and 50, respectively, which were significantly higher than those of the unimmunized control animals (*p* < 0.0001). Animals vaccinated with the SARS-CoV-2 PsIV at 10^10^ particles/dose elicited neutralizing antibodies against the Washington strain with an MN_80_ GMT of 14 and a *p* value < 0.0001 when compared to the controls but did not elicit any significant immune response against the heterologous Delta strain (MN_80_ GMT of 3, *p* > 0.05 vs. controls). Interestingly, the prime-boost group animals elicited the highest neutralizing antibody titers against both the Washington strain and the Delta variant strains, with MN_80_ GMTs of 698 and 320, respectively (*p* < 0.0001 vs. controls). Therefore, dose-dependent neutralizing antibody responses against the Washington and Delta strains were observed (Figure 3) for the SARS-CoV-2 PsIV in the NHPs.

**Figure 4:**
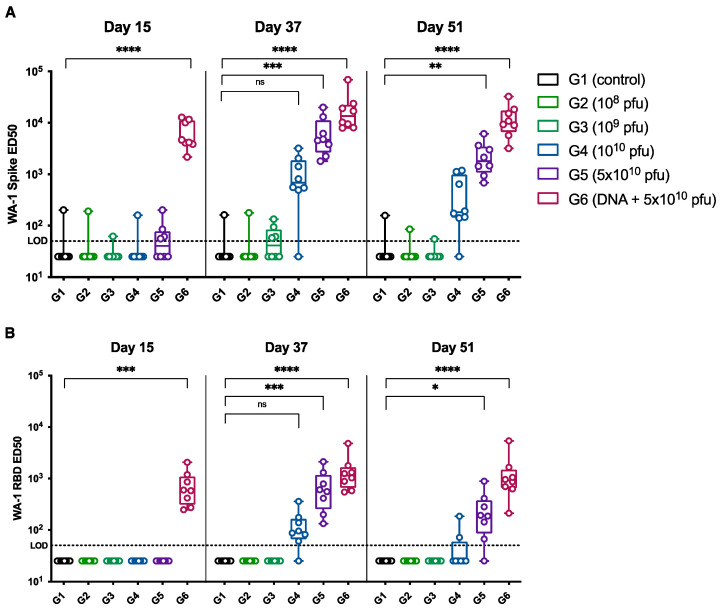
ED50 of SARS-CoV-2 spike- (**A**) and RBD-specific (**B**) IgG antibodies in the NHPs sera 15 days after administration of the first dose (day 15), 7 days after the second dose of vaccination (day 37) and 21 days after the second dose (day 51). Average of duplicate values for each sample is shown. Statistical comparison of ED50 values were performed using Kruskal–Wallis test for each timepoint. *p*-values were adjusted for multiple testing using Dunn’s multiple comparison test. ns: no significance, * *p* ≤ 0.05, ** *p* ≤ 0.01, *** *p* ≤ 0.001, **** *p* ≤ 0.0001.

We also measured the SARS-CoV-2 spike-protein-specific IgG and RBD-specific IgG antibody levels in the sera at different timepoints after vaccination. The spike-protein-specific IgG levels measured at day 15 (two weeks after the first dose), day 37 (seven days after the second dose) and day 51 (twenty-one days after the second dose) are shown in Figure 4A. Fourteen days after the first dose of the SARS-CoV-2 PsIV, there was no detectable level of spike-protein-specific IgG, as seen for the day 15 sera. However, after the second dose of the SARS-CoV-2 PsIV, a dose-dependent increase in the spike-protein-specific IgG antibody levels was observed in the day 37 and day 51 sera. Consistent with the neutralizing antibody levels observed after the SARS-CoV-2 PsIVs, lower doses (10^8^ and 10^9^ particles doses) did not produce a detectable level of spike-protein-specific IgG even after the administration of the second doses, whereas the higher doses (10^10^ and 5 × 10^10^ particles doses) exhibited a dose-dependent increase in the spike-protein-specific IgG levels after the second dose of the vaccinations. Consistent with the neutralizing antibody titers, the prime-boost group produced the highest spike-protein-specific serum IgG levels. A dose-dependent increase in the RBD-specific IgG antibody levels was also observed at different timepoints after the administration of the second dose of the SARS-CoV-2 PsIV, as shown in Figure 4B.

**Figure 5:**
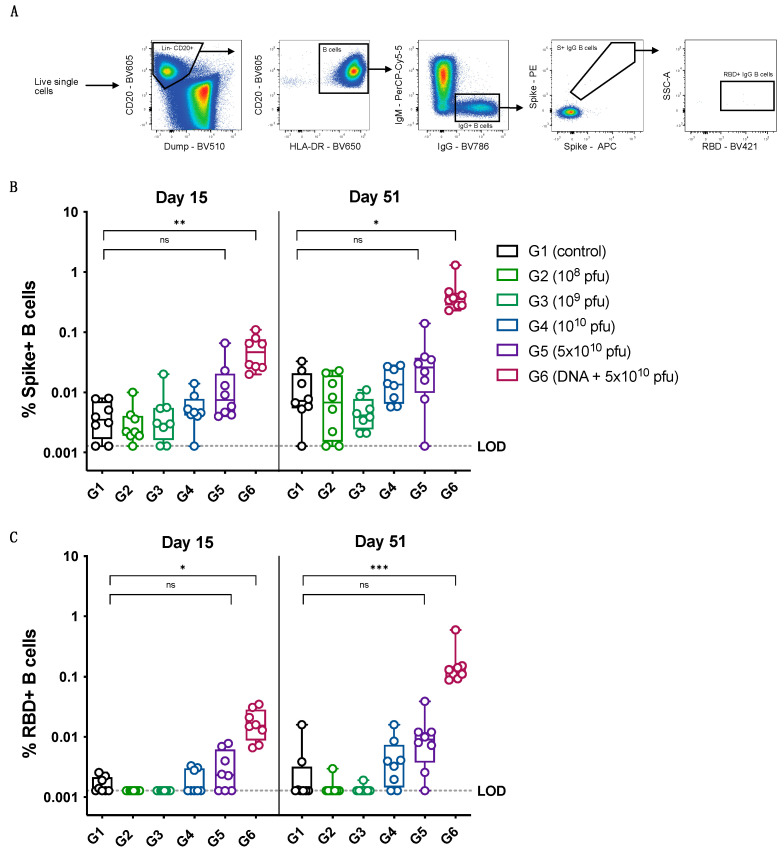
Memory B cell probing. (**A**) Gating strategy to define spike- and RBD-specific IgG memory B cells using flow cytometry. (**B**) Percentage of spike-positive IgG+ B cells of total B cells (LOD = 0.00128%). (**C**) Percentage of RBD-positive IgG+ B cells of total B cells (LOD = 0.00128%). Statistical comparison was performed using Kruskal–Wallis test for each timepoint. *p*-values were adjusted for multiple testing using Dunn’s multiple comparison test. ns: no significance, * *p* ≤ 0.05, ** *p* ≤ 0.01, *** *p* ≤ 0.001.

**Figure 6:**
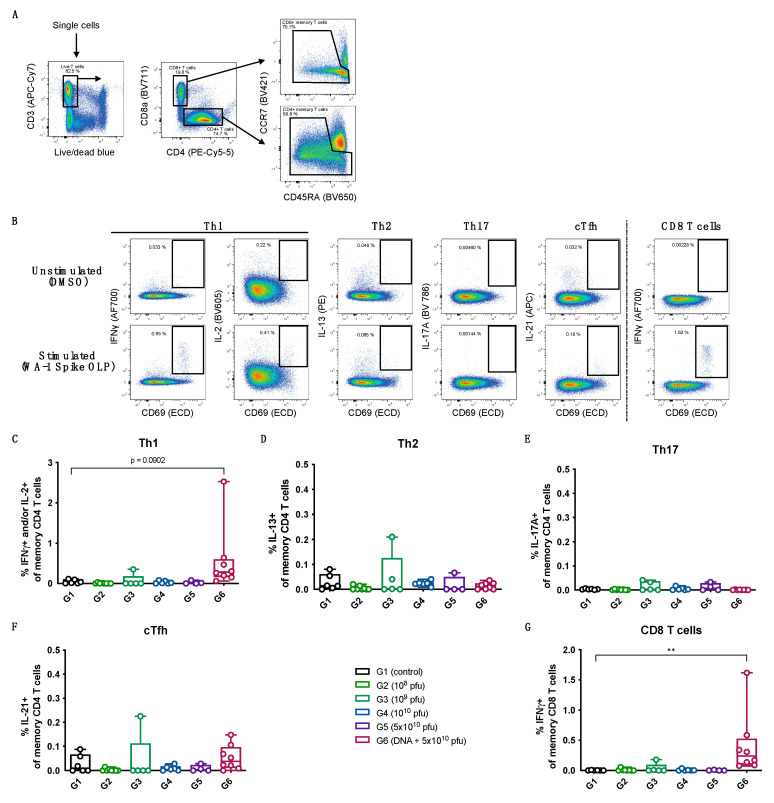
Memory T cell recall assay. (**A**) Gating strategy to define CD4 and CD8 memory T cells. (**B**) Example of T cell responses in response to no stimulation (DMSO) or stimulation with WA-1 spike-derived overlapping peptide (OLP) library using PBMCs collected 7 days after administration of the second dose (day 37). A sample from a group 6 animal is presented. Percentage of spike-specific Th1 (**C**), Th2 (**D**), Th17 (**E**), circulating Tfh (**F**) CD4 memory T cells and CD8 memory T cells (**G**) 7 days after administration of the second dose (day 37). All data are background-subtracted using unstimulated condition (DMSO). Statistical comparison between groups was performed using Kruskal–Wallis test. *p*-values were adjusted for multiple testing using Dunn’s multiple comparison test. ** *p* ≤ 0.01.

A similar trend of a dose-dependent increase in the levels of memory B cells specific to the spike protein as well as the RBD was observed for the vaccinated animals, with the prime-boost group exhibiting the highest levels of memory B cells at day 15 (after the first dose of vaccinations) and at day 51 (after the last dose of vaccinations), as shown in Figure 5. The spike-protein-specific and RBD-specific memory B-cell data (Figure 5B,C) show a good correlation with the spike-specific and RBD-specific IgG levels measured for the different vaccine dose groups (Figure 4A,B). The T cell responses to the spike protein were measured at day 37 (7 days after the second immunization) using a recall assay with an overlapping peptide library covering the full spike protein. The animals in the groups immunized with the PsIV alone did not have detectable spike-specific T cell responses; however, the prime-boost group showed a clear induction of spike-specific Th1 responses, as well as potent CD8 T cell responses (Figure 6).

**Figure 7:**
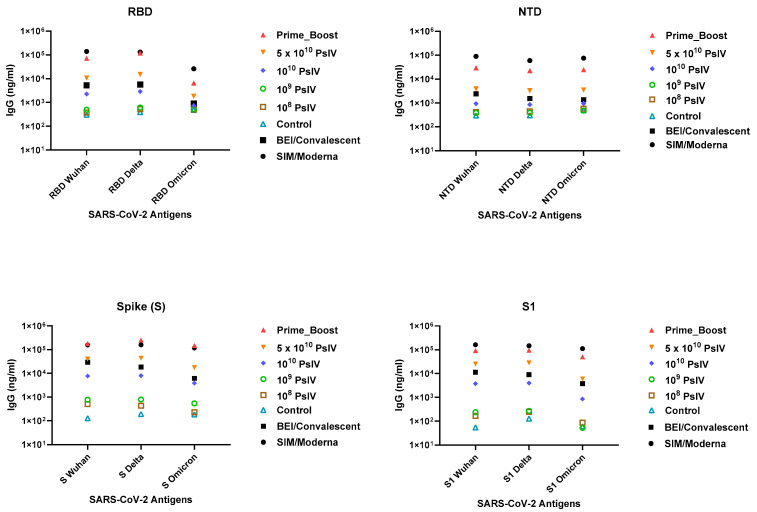
Antibody titers (average of triplicate results) against various spike protein domains of SARS-CoV-2 variants in day 51 sera (21 days after the second dose). BEI convalescent sera were used as the reference sera. SIM/Moderna sera were used as positive controls.

The serum IgG titers against various spike protein domains (RBD, N-terminal and S1 domains) of the SARS-CoV-2 Wuhan, Delta and Omicron strains were measured (Figure 7). Consistent with the observed neutralizing antibody levels, a dose-dependent increase in the IgG levels against various spike protein domains of different variants were observed. Interestingly, the antibody levels elicited by the prime-boost group and the highest PsIV dose were higher than the antibody levels elicited by the reference convalescent sera obtained from BEI. These data suggest that the SARS-CoV-2 PsIV is highly immunogenic and elicits a dose-dependent neutralizing antibody response, while the heterologous prime-boost regimen of two doses of a DNA vaccine and a booster dose of the SARS-CoV-2 PsIV elicited the highest neutralizing antibody levels.

### 3.3. Protection against Infection by the SARS-CoV-2 Delta Variant

**Figure 8:**
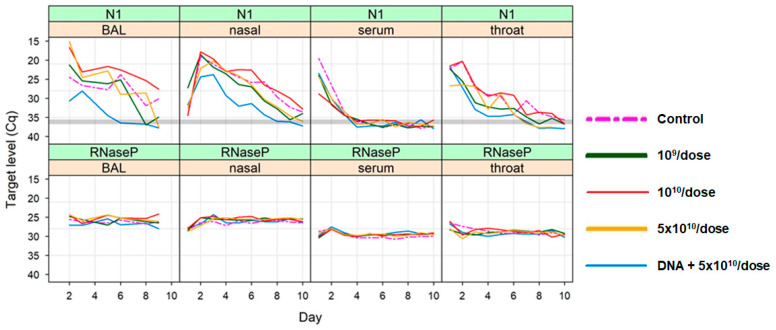
Summary of qRT-PCR results for BAL, nasal swab and throat swab samples collected after the challenge with SARS-CoV-2 Delta strain. Average of triplicate values for each RT-PCR reaction is shown. SARS-CoV-2-specific RNA was amplified using the primers and probes for nucleocapsid protein gene (indicated as N1). RNAse P gene amplification (indicated as RNAseP) served as the internal control.

**Figure 9:**
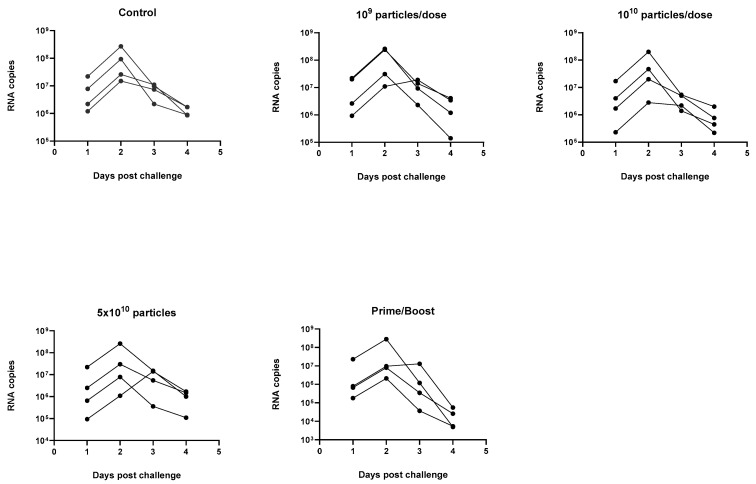
RNA copy numbers in nasal swab samples collected after the challenge with SARS-CoV-2 Delta strain using digital droplet RT-PCR (average of triplicate values for each RT-ddPCR reaction is shown here).

The ability of the SARS-CoV-2 PsIV to protect against infection by the SARS-CoV-2 Delta variant in the NHPs was evaluated by challenging the animals 60 days after the administration of the last dose of the vaccine. Briefly, four animals from the control group (Group 1), four animals from each of the groups that received higher doses of the SARS-CoV-2 PsIV (groups 3–5) and four animals from the prime-boost group (group 6) were moved to ABSL-3 on day 103 and challenged with 2 × 10^5^ PFU of the SARS-CoV-2 Delta variant at a dose of 0.5 mL on day 111, via intranasal instillation. The animals were observed for symptoms of COVID-19 illness for up to 14 days after the challenge, and nasal and throat swabs were collected from all the animals for up to 14 days after the challenge. BAL samples were also collected from all the animals until day 9 after the challenge. All the samples were analyzed using quantitative RT-PCR and digital droplet RT-PCR to determine the SARS-CoV-2 titers, and the results are shown in Figure 8 (quantitative RT-PCR results) and Figure 9 (digital droplet RT-PCR results).

SARS-CoV-2 RNA copy numbers of 10^4^–10^8^ per mL in the BAL samples of the control animals were observed on days 1–9 after the challenge (BAL samples were not collected after day 9). RNA copy numbers of 10^4^–10^6^ per mL were also observed in the BAL samples from the animals in the prime-boost group on days 2 and 3 after the challenge but dropped to below 10^4^ copies per mL by day 5 after the challenge and were undetectable after that. The BAL samples from the animals vaccinated with 5 × 10^10^ particles of the PsIV showed a marked reduction in their SARS-CoV-2 RNA copy numbers 6–9 days after the challenge, although it was not as pronounced as the reduction observed in the prime-boost-vaccinated animals. These results suggest that the heterologous prime-boost vaccination regimen protected the animals from SARS-CoV-2 infection, and a dose-dependent protection against infection was observed for the PsIV. Similar results were observed for the nasal and throat swab samples, as discussed below. The peak SARS-CoV-2 RNA copy number values for the throat swab samples from the prime-boost group were observed on day 1 and dropped significantly within 3 days after the challenge (below 10^3^ copies per mL). Similarly, the nasal swab RNA copy numbers for the prime-boost animals peaked on day 2–3 after the challenge and dropped to values below 10^3^ copies per mL by day 7 after the challenge. The throat swab RNA copy numbers for the animals in group 5 (5 × 10^10^ particles of PsIV) were observed on day 2–3 and dropped significantly within 6 days after the challenge (below 10^3^ copies per mL). Similarly, the nasal swab RNA copy numbers for the animals in group 5 (5 × 10^10^ particles of PsIV) peaked on day 3 post-challenge and dropped to values below 10^4^ copies per mL by day 8.

Analysis of the BAL, nasal and throat swab samples collected after the challenge with SARS-CoV-2 Delta virus show that the animals that received the heterologous prime-boost vaccinations were able to quickly clear SARS-CoV-2 after the challenge, as shown in Figure 8 and Figure 9. Although reduced viral titers, as indicated by the SARS-CoV-2 RNA copies, were observed in the samples from the animals vaccinated with the highest dose of the SARS-CoV-2 PsIV when compared to the control animals, the prime-boost vaccination provided the best overall protection against infection by SARS-CoV-2.

## 4. Discussion

A high rate of SARS-CoV-2 transmission caused the global emergence of COVID-19 pandemic, resulting in global healthcare and economic crises. Although the rapid development and approval of SARS-CoV-2 vaccines led to a massive reduction in COVID-19-related hospitalization and death, the pandemic is far from over due to rapidly emerging immune escape variants leading to reduced vaccine effectiveness. Therefore, the development of a broadly protective COVID-19 vaccine remains a global priority. Unlike the mRNA and subunit vaccines developed to produce an immune response against a single antigen, such as the spike protein, a whole-virus inactivated vaccine can provide broader protection by inducing immune responses against several epitopes to cover multiple proteins, including membrane, envelope and nucleocapsid proteins [13,24]. Here, we prepared a highly purified psoralen-inactivated whole-virus COVID-19 vaccine and performed preclinical immunogenicity and protective efficacy studies in NHPs. A psoralen-inactivated vaccine was developed to target multiple epitopes of SARS-CoV-2 antigens, while most COVID-19 vaccines target only the spike glycoprotein. A psoralen UV-irradiation-based vaccine is expected to preserve the surface proteins in their native conformation, whereas β-propiolactone and formalin inactivation of viruses is expected to alter both the nucleic acid and the surface protein structures. Therefore, we expected the SARS-CoV-2 PsIV to elicit better immune responses than β-propiolactone- or formalin-inactivated vaccines. Previously, we have shown that a psoralen-inactivated dengue virus vaccine elicited superior immune responses in NHPs compared to formalin-inactivated dengue vaccines [19]. Therefore, the development and evaluation of a psoralen-inactivated SARS-CoV-2 vaccine with the potential to broadly protect against SARS-CoV-2 variants of concern were a logical approach.

Previously, we evaluated the immunogenicity of an alum-adjuvanted and an Advax-CpG-adjuvanted SARS-CoV-2 PsIV in mice. The results from our study in mice indicated that the Advax-CpG-adjuvanted PsIV elicited significantly better immune responses than the alum-adjuvanted PsIV, consistent with the data obtained for other viral vaccines in small animal models [25]. Furthermore, the results from our mouse study suggested that an Advax-CpG-adjuvanted PsIV induces a strong Th1 response that may help prevent the vaccine-related lung immunopathology found to be associated with excess Th2 bias observed for other inactivated whole-virus vaccines [3]. Therefore, in this study, we used the Advax-CpG adjuvant to evaluate the immunogenicity of a SARS-CoV-2 PsIV in NHPs.

We acknowledge several limitations that warrant further discussion. First, our claim that the virus-based vaccine elicits broader protection in targeting different viral proteins was primarily supported by examining the binding affinity of the antibodies to the S protein. Unfortunately, due to resource constraints, we were unable to test the samples against other SARS-CoV-2 antigens during the performance period of this study. We aim to address this gap in future projects to provide more comprehensive evidence of the vaccine’s breadth of protection. Second, our findings in the current study differ from those in our previous mouse study, particularly regarding the vaccine’s ability to elicit T cell and B cell responses. This discrepancy may be attributed to the cell viability issues encountered during the T cell and B cell assays in the current study, which were not present in the mouse study. We plan to further investigate and refine our assay methodologies to overcome these challenges in future research. Lastly, the potential of the SARS-CoV-2 PsIV as a booster for individuals previously vaccinated with mRNA vaccines remains an intriguing hypothesis. Due to the unavailability of mRNA vaccines for research use during the study period, we were unable to directly test this hypothesis. As mRNA vaccines become more accessible for research, we look forward to exploring a prime-boost regimen involving our SARS-CoV-2 PsIV and mRNA vaccines to evaluate their combined protective efficacy against circulating variants.

Here, we observed that the Advax-CpG-adjuvanted PsIV elicited a dose-dependent neutralizing antibody response against both the Washington and Delta strains. As evidenced by the RNA copy numbers observed in the BAL samples, as well as the nasal and throat swab samples from the PsIV-vaccinated animals challenged with the Delta variant, higher doses of the PsIV (equivalent to 10^10^ and 5 × 10^10^ particles/dose) provided the best protection against SARS-CoV-2 infection. Interestingly, the animals vaccinated with the heterologous prime-boost regimen (two doses of a DNA vaccine followed by a booster dose of the PsIV) generated the highest neutralizing antibody responses and were able to quickly clear SARS-CoV-2 when challenged with the Delta strain. The prime-boost group exhibited a strong Th1-polarized CD4 T cell response and an induction of spike-specific CD8 T cell responses. Given that the licensed COVID-19 mRNA vaccines generate poor CD8 T cell responses, priming with DNA vaccines may be an alternative strategy for the induction of antigen-specific CD8 T cells [26]. We also observed a dose-dependent increase in spike-protein-specific serum IgG levels for the PsIV. Again, the sera from the animals vaccinated with the prime-boost strategy exhibited the highest spike-protein-specific serum IgG concentration. These results indicate that a two-dose vaccination schedule, even at the highest concentration of the PsIV tested in this study (5 × 10^10^ particles/dose), may not be sufficient to elicit an immune response and may require the administration of a third dose for the maturation of vaccine-specific B cells to achieve the best immune responses [20,27]. In this study, we were limited to using 5 × 10^10^ particles/dose as the highest dose since we were not able to generate higher titers of the virus in the laboratory. Therefore, an increase in the SARS-CoV-2 PsIV dose to 5 × 10^11^ particles or more in future studies is warranted to see whether this would provide more complete protection from SARS-CoV-2 infection. We are currently working with a commercial vendor to make higher titers of the SARS-CoV-2 PsIV under current good manufacturing practices (cGMP) conditions for a future study to evaluate higher doses of the vaccine, including 5 × 10^11^ particles per dose and an anticipated phase 1 clinical evaluation. As mentioned earlier, the prime-boost regimen using two doses of the DNA vaccine followed by a third dose of the SARS-CoV-2 PsIV induced the highest neutralizing antibody response and completely protected the animals from infection when they were challenged with the Delta strain. Therefore, the SARS-CoV-2 PsIV has great potential to be used as a booster shot for already vaccinated people to prevent infection by newly emerging variants, including the currently circulating Omicron variant.

## 5. Patents

NMRC filed a non-provisional patent titled “Psoralen-inactivated Coronavirus Vaccine and Method of Preparation” on 1 December 2021.

## Figures and Tables

**Table 1:** Dosage for SARS-CoV-2 PsIV evaluation in nonhuman primates.

Groups	Vaccine Formulation	SARS-CoV-2 PsIV Dose (Expressed as Spike Protein Equivalent) Given
Group 1	Advax-CpG + PBS (Controls) (Days 0 and 30)	not applicable
Group 2	SARS-CoV-2 PsIV + Advax-CpG (Days 0 and 30)	0.0075 µg of spike protein/dose
Group 3	SARS-CoV-2 PsIV + Advax-CpG (Days 0 and 30)	0.075 µg of spike protein/dose
Group 4	SARS-CoV-2 PsIV + Advax-CpG days 0 and 30)	0.750 µg of spike protein/dose
Group 5	SARS-CoV-2 PsIV + Advax-CpG (Days 0 and 30)	3.75 µg of spike protein/dose
Group 6	Plasmid DNA (Days −30 and 0) SARS-CoV-2 PsIV + Advax-CpG (Day 30)	5 mg DNA/dose and 3.75 µg of spike protein/dose

## Data Availability

The original contributions presented in the study are included in the article/Supplementary Materials; further inquiries can be directed to the corresponding author.

## Supplementary Materials

The following supporting information can be downloaded at: https://www.mdpi.com/article/10.3390/vaccines12050451/s1. 1. Western Blot analysis of SARS-CoV-2 PsIV vaccine, after the two-step chromatographic purification using cellufine MAX-DexS-VirS column and a Capto Core 700 column. Presence of SARS-CoV-2 spike glycoprotein (S) was confirmed using anti-spike protein antibody as the primary antibody. Presence of SARS-CoV-2 nucleocapsid protein (N) was confirmed using anti-nucleoprotein protein antibody as the primary antibody. 2. Western Blot analysis of SARS-CoV-2 PsIV vaccine, after the two-step chromatographic purification using cellufine MAX-DexS-VirS column and a Capto Core 700 column. Presence of SARS-CoV-2 membrane protein (M) was confirmed using anti-membrane protein antibody as the primary antibody.

## Disclaimer

The views expressed in this article reflect the research conducted by the authors and do not necessarily reflect the official policy or position of the Department of the Navy, the Department of Defense or the U.S. Government. Copyright Statement: The authors are military service members or federal/contracted employees of the U.S. Government. This work was prepared as part of their official duties. Title 17 U.S.C. § 105 provides that ‘Copyright protection under this title is not available for any work of the U.S. Government’. Title 17 U.S.C. § 101 defines a U.S. Government work as work undertaken by military service members or employees of the U.S. Government as part of those people’s official duties.
