# Supplementary material for: Immunogenicity and Protective Efficacy of Psoralen-Inactivated SARS-CoV-2 Vaccine in Nonhuman Primates
Source: Vaccines (Basel). 2024 Apr 24;12(5):451. doi: 10.3390/vaccines12050451 (PMC11125875; doi:10.3390/vaccines12050451)

Western Blot analysis of SARS-CoV-2 PsIV vaccine, after the two-step chromatographic purification using cellufine MAX-DexS-VirS column and a Capto Core 700 column. Presence of SARS-CoV-2 spike glycoprotein (S) was confirmed using anti-spike protein antibody as the primary antibody. Presence of SARS-CoV-2 nucleocapsid protein (N) was confirmed using anti-nucleoprotein protein antibody as the primary antibody.

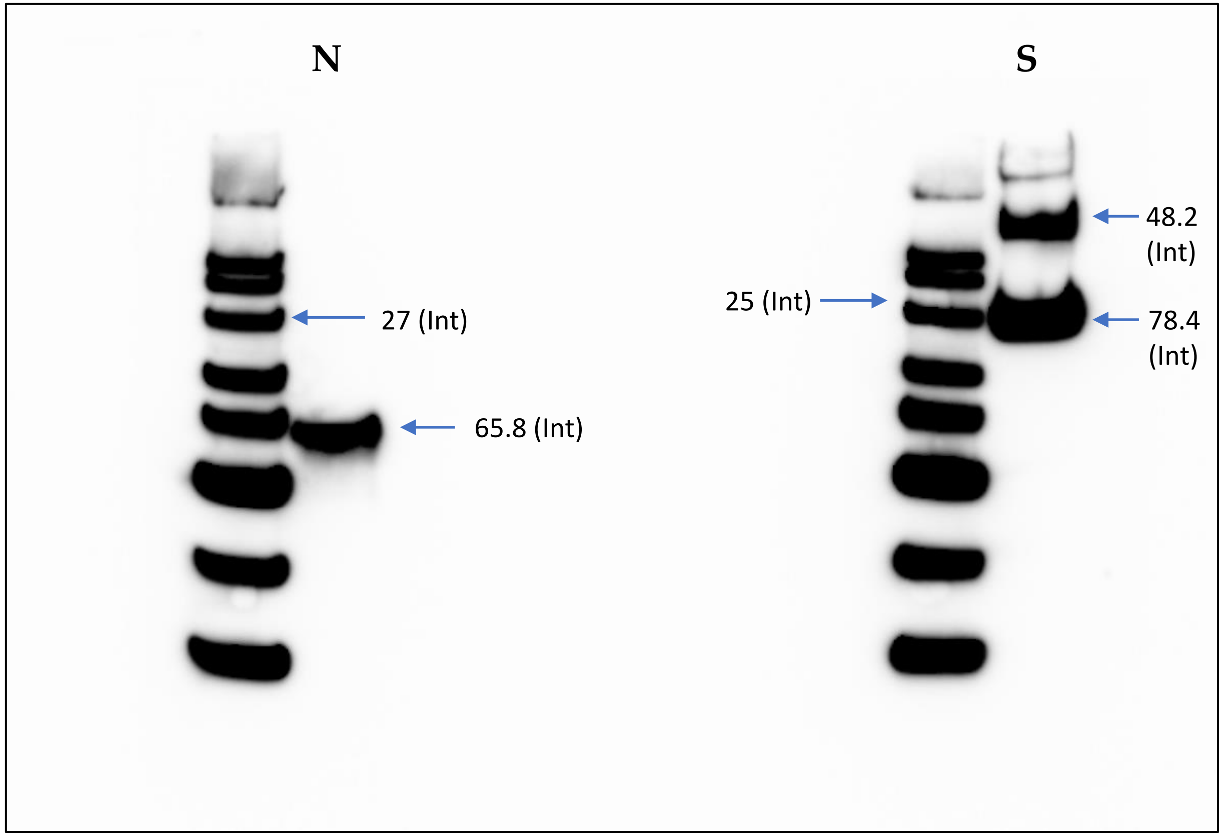

Western Blot analysis of SARS-CoV-2 PsIV vaccine, after the two-step chromatographic purification using cellufine MAX-DexS-VirS column and a Capto Core 700 column. Presence of SARS-CoV-2 membrane protein (M) was confirmed using anti-membrane protein antibody as the primary antibody.

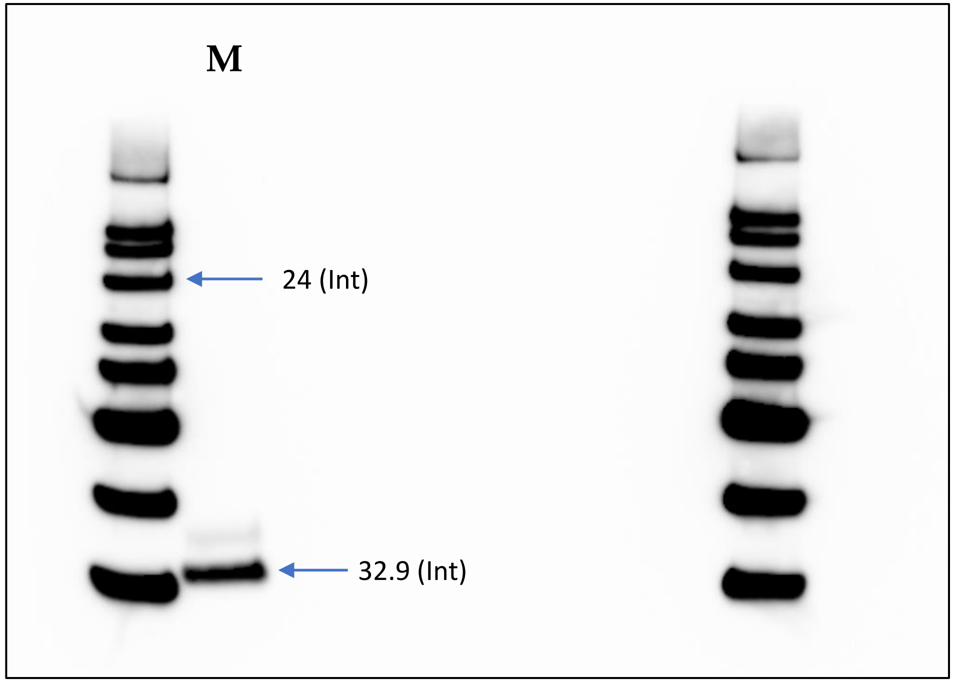

Supplement: Supplementary file 1 [file vaccines-12-00451-s001.zip › vaccines-2866775-supplementary.pdf]
